# Targeting mannose receptor expression on macrophages in atherosclerotic plaques of apolipoprotein E-knockout mice using ^68^Ga-NOTA-anti-MMR nanobody: non-invasive imaging of atherosclerotic plaques

**DOI:** 10.1186/s13550-019-0474-0

**Published:** 2019-01-21

**Authors:** Zohreh Varasteh, Sarajo Mohanta, Yuanfang Li, Nicolás López Armbruster, Miriam Braeuer, Stephan G. Nekolla, Andreas Habenicht, Hendrik B. Sager, Geert Raes, Wolfgang Weber, Sophie Hernot, Markus Schwaiger

**Affiliations:** 1 Department of Nuclear Medicine, Klinikum rechts der Isar der TUM, Ismaninger-Strasse 22, 81675 Munich, Germany; 2 Institute for Cardiovascular Prevention, University Hospital of Ludwig-Maximilians-University, Munich, Germany; 3 Deutsches Herzzentrum München, Klinik für Herz und Kreislauferkrankungen, Technical University of Munich, Munich, Germany; 4 Department of Bio-engineering Sciences, Vrije Universiteit Brussel, Brussels, Belgium; 5 In Vivo Cellular and Molecular Imaging (ICMI), Vrije Universiteit Brussel, Brussels, Belgium

**Keywords:** Atherosclerotic plaques, Non-invasive imaging, Inflammation, Alternatively differentiated macrophages, Mannose receptor, PET/CT

## Abstract

**Background:**

Rupture-prone atherosclerotic plaques are characterized by heavy macrophage infiltration, and the presence of certain macrophage subsets might be a sign for plaque vulnerability. The mannose receptor (MR, CD206) is over-expressed in several types of alternatively activated macrophages. In this study, our objective was to evaluate the feasibility of a Gallium-68 (^68^Ga)-labelled anti-MR nanobody (^68^Ga-anti-MMR Nb) for the visualization of MR-positive (MR^+^) macrophages in atherosclerotic plaques of apolipoprotein E-knockout (ApoE-KO) mice.

**Results:**

NOTA-anti-MMR Nb was labelled with ^68^Ga with radiochemical purity > 95%. In vitro cell-binding studies demonstrated selective and specific binding of the tracer to M2a macrophages. For in vivo atherosclerotic plaque imaging studies, ^68^Ga-NOTA-anti-MMR Nb was injected into ApoE-KO and control mice intravenously (i.v.) and scanned 1 h post-injection for 30 min using a dedicated animal PET/CT. Focal signals could be detected in aortic tissue of ApoE-KO mice, whereas no signal was detected in the aortas of control mice. ^68^Ga-NOTA-anti-MMR Nb uptake was detected in atherosclerotic plaques on autoradiographs and correlated well with Sudan-IV-positive areas. The calculated ratio of plaque-to-normal aortic tissue autoradiographic signal intensity was 7.7 ± 2.6 in aortas excised from ApoE-KO mice. Immunofluorescence analysis of aorta cross-sections confirmed predominant MR expression in macrophages located in the fibrous cap layer and shoulder region of the plaques.

**Conclusions:**

^68^Ga-NOTA-anti-MMR Nb allows non-invasive PET/CT imaging of MR expression in atherosclerotic lesions in a murine model and may represent a promising tool for clinical imaging and evaluation of plaque (in)stability.

## Background

Atherosclerosis is a leading cause of morbidity and mortality worldwide [1]. Despite substantial advances in understanding and treatment of conventional risk factors, the prevalence of diseases associated with atherosclerosis keeps growing. It remains clinically challenging to identify asymptomatic individuals at high risk for developing acute complications of atherosclerosis. A main focus has therefore been assigned to develop non-invasive imaging approaches for plaque characterization. Radioisotope-based molecular imaging has emerged at the forefront of methods for identifying biological aspects of atherosclerotic plaques and assessing hallmarks involved in plaque vulnerability, which are not possible to capture with morphometric imaging. However, the selection of a suitable molecular target in rupture-prone plaques remains a major challenge with this approach [2].

Monocyte-derived macrophages are the first inflammatory cells to invade atherosclerotic lesions and are recognized as key pathophysiologic agents in atherosclerosis [3–5]. They are involved at multiple stages of plaque development and are therefore increasingly gaining importance as imaging targets in atherosclerosis. However, macrophages are a heterogeneous population of cells, and different subsets could be either pro- or anti-atherosclerotic [6, 7]. Distinct macrophage phenotypes can be assessed by the expression of different surface biomarkers and chemokine receptors [6–11].

Certain macrophage subtypes express carbohydrate-binding receptors, e.g., mannose receptor (MR, CD206) [6, 7], which is a highly effective endocytic C-type lectin receptor (175 kDa). High expression of MR was reported on macrophages located in the fibrous cap of plaques, whereas apoptotic macrophages of the lipid core have shown only low expression [12]. Therefore, effective targeting of macrophages using MR-specific radioconjugates is a potential approach for imaging atherosclerotic plaques.

Recently, nanobodies (Nbs) against macrophage mannose receptor (MMR) have been developed, and their potential as in vivo diagnostic tracers for non-invasive imaging a subpopulation of tumour-infiltrating macrophages [13, 14] and joint inflammation in rheumatoid arthritis [15] have been well documented.

Nbs, which are derived from camelid heavy chain-only antibodies, are the smallest available antigen-binding fragments [16]. Their small size (~ 15 kDa) is favourable for rapid localization at the target tissue and clearance from circulation via kidneys, which results in high target-to-background signal ratios in a short time [16]. Consequently, imaging with Nb-based radioconjugates can be carried out as early as 1 h post-injection (p.i.), enabling the use of short-lived radioisotopes, e.g., Gallium-68 (^68^Ga).

In the present study, our objective was to evaluate the potential of ^68^Ga-NOTA-anti-MMR Nb for selectively targeting MR-positive (MR^+^) macrophages and non-invasively imaging atherosclerotic plaques. This approach might pave the way for better understanding the role of MR^+^ macrophages in plaque progression and rupture in patients. After investigating the in vivo biodistribution and specificity of ^68^Ga-NOTA-anti-MMR Nb in wildtype mice, a thorough assessment as a tracer for non-invasive in vivo nuclear molecular imaging of atherosclerotic lesions was performed in apolipoprotein E-knockout (ApoE-KO) mice.

## Methods

### Animal model

For biodistribution and in vivo specificity studies, female C57BL/6 mice (5 weeks old, 18–21 g weight, from Charles River Laboratories) referred to as wildtype were used. Adult ApoE-KO mice (Apoe^tm1Unc^, female, 28 weeks old, 28–32 g weight, from Jackson laboratory) were used for in vivo and ex vivo imaging studies. ApoE-KO mice were on high fat diet (42% calories from fat, E 15721-347 from ssniff Spezialdiäten GmbH) for 20 weeks. Age-matched female C57BL/6 mice were used as controls. Control mice were fed a normal chow diet.

### Preparation of the radiotracer

The anti-MMR Nb 3.49, cross-reactive for both the mouse (K_D_ = 12 nM) and human (K_D_ = 1.8 nM) homologue of MR [14], was conjugated with the bifunctional chelator 2-S-(4-isothiocyanatobenzyl)-1,4,7-triazacyclononane-N,N′,N″-triacetic acid (p-SCN-Bn-NOTA, Macrocyclics) as described elsewhere [17]. A solution of NOTA-anti-MMR Nb was labelled with ^68^Ga at room temperature. Briefly, 25 μl (55.25 μg) of NOTA-anti-MMR Nb solution was incubated with 2.275 ml (~ 400 MBq) of ^68^Ga eluate (in 0.05 M HCl) and 200 μl of sodium acetate buffer (2 M, pH 5) for 15 min. Radiochemical purity (RCP) of the tracer was determined by radio-instant thin layer chromatography (radio-ITLC) using 0.1 M sodium citrate (pH 5) as the mobile phase.

For further animal studies, the radiotracer was purified using a PD-10 column (GE Healthcare) preconditioned with 25 ml of phosphate-buffered saline (PBS).

### In vitro binding specificity assay

Fresh peripheral blood mononuclear cells (PBMC) were isolated from healthy human donor blood using standard Ficoll-Paque density-gradient (Amersham Biosciences). CD14-positive monocytes were isolated from PBMCs using positive selection technology (MACS technology; Miltenyi Biotec). Isolated cells were plated in 12-well dishes (10^6^ cells/dish) for attachment using Monocyte Attachment Medium, according to the manufacturer’s protocol (C28051, PromoCell). Subsequent differentiation was performed by incubating the cells with M1 or M2-Macrophage Generation Medium DXF (C-28055 or C-28056, PromoCell). M1-activation of macrophages was achieved by addition of IFN-ɣ (50 ng/ml, C-60724, PromoCell) and LPS (100 ng/ml, Sigma-Aldrich), and M2a-activation of M2-macrophages was achieved by addition of 20 ng/ml IL-4 stimulatory factor, according to manufacturer’s protocol (C-61420A, PromoKine). Differentiated cells were incubated with 10 nM ^68^Ga-NOTA-anti-MMR Nb for 30 min at room temperature in order to avoid receptor internalization. One set of M2a dishes was co-incubated with a 1000-fold excess amount of non-labelled NOTA-anti-MMR Nb referred as M2a-blocked. After incubation, media were removed from cell dishes. Cells were washed and detached using Macrophage Detachment Solution DXF (C-41330, PromoCell). Cell solutions were transferred into fraction tubes. A fraction of cell suspensions was used for cell counting. The radioactivity of the remaining cells was measured in an automated gamma counter (PerkinElmer 2480 WIZARD^2^). The uptake was calculated as cell-associated radioactivity.

### Biodistribution and in vivo binding specificity studies

Two groups (*n* = 5 per group) of wildtype mice were injected intravenously (i.v.) with 8–10 MBq of ^68^Ga-NOTA-anti-MMR Nb (3–4 μg) to assess blood clearance and overall biodistribution of the tracer. One group was co-injected with a 100-fold excess amount of non-labelled NOTA-anti-MMR Nb and are referred to as the blocked group. The mice were sacrificed by a high dose of Pentobarbital (Narcoren) 1 h p.i. Blood was collected by heart puncture and organs and tissues were excised. The samples were put in pre-weighed plastic vials. The samples were weighed, and their radioactivity was measured in the gamma counter against a standard of known activity. The uptake in tissue and organs was calculated as percentage of injected activity per gram of tissue (% IA/g) corrected for decay. For the gastrointestinal tract and the carcass, percentage of injected activity per gram of whole sample was calculated (% IA/g).

### In vivo imaging (PET/CT study)

Two groups of mice, ApoE-KO (*n* = 15) and control (*n* = 6), were used for in vivo and ex vivo imaging studies. Mice were kept fully sedated with 1.5–2% isoflurane during injections and PET/CT imaging. Images were acquired using the Inveon small animal PET/CT scanner (Siemens, Knoxville, TN, USA) 1 h after i.v. injection of ^68^Ga-NOTA-anti-MMR Nb (8–10 MBq, 3–4 μg). Briefly, CT anatomic images were acquired (80 kV, 500 μA) with a pixel size of 0.1 mm. After CT imaging, static PET images were acquired with an acquisition time of 20 min. Images were reconstructed as single frames using Siemens Inveon software, employing a 3-dimensional ordered subsets expectation maximum algorithm (OSEM3D) without scatter and attenuation correction. A group of ApoE-KO mice (*n* = 4), referred to as the ApoE-KO blocked group, was co-injected with blocking dose (100-fold excess) of non-labelled NOTA-anti-MMR Nb. For quantification of vascular uptake, circular regions of interest (ROIs) were placed on axial PET/CT images of the thoracic and abdominal aortas and signal intensities were recorded as kBq/cc. ROIs were identified by the same person with their centres at the point of local maximum ^68^Ga-NOTA-anti-MMR Nb uptake.

### Ex vivo imaging (autoradiography and Sudan-IV staining)

After PET/CT scans, mice were euthanized with an overdose of isoflurane. The whole length of the aortas (from the sinotubular junction to the iliac bifurcation) was excised using a dissection microscope (Zeiss Stemi DV4 SPOT) [18]. The radioactivity of the dissected aortic tissues was measured in the gamma counter. After radioactivity measurements, the adventitial tissue on the aortas was removed by careful dissection. The aortas of ApoE-KO non-blocked (*n* = 9), ApoE-KO blocked (*n* = 4) and control (*n* = 4) mice were kept intact and longitudinally exposed to phosphor imaging plates (Fuji Imaging Plate, Fujifilm). Radioactivity signals were collected for 1 h. The imaging plates were scanned, autoradiographs were obtained with a phosphor imaging system (Raytest, Straubenhardt, Germany) and images were analysed for count densities. ROIs were placed on the whole aorta to measure total quantum level (QL) units contained in that area. In addition, ROIs were placed over normal aortic tissue to calculate signal intensities per unit area (QL/mm^2^) of normal aortic tissue.

After autoradiography, aortas were opened longitudinally, mounted en face on a black wax surface to expose the luminal side and stained with Sudan-IV for neutral lipids using a previously published method [18]. The images of en face stained aortas were used to measure the whole aorta and lesional surface areas. Briefly, the outer border of the whole aortas as well as each Sudan-IV stained plaque surface was encircled manually and their areas (mm^2^) were measured using the public domain software ImageJ (National Institutes of Health (NIH), USA) [18]. Data were used to calculate autoradiographic signal intensity (QL/mm^2^) in the whole aorta, in normal aortic tissue and in plaques.

### Immunofluorescence staining and confocal microscopy

For immunofluorescence staining, 10-μm-thick cross-sections were prepared from excised aortas of ApoE-KO (*n* = 2) and control (*n* = 2) mice. Parallel sections were immunostained as previously described [19]. Briefly, slides were fixed with acetone, rehydrated in PBS, blocked with 10% donkey serum and incubated for 3 h with primary antibodies diluted with 2.5% bovine serum albumin (BSA). Primary antibodies include rat anti-mouse CD68 (clone FA-11, Bio-Rad) for macrophages and goat anti-mouse CD206 antibody (clone MR5D3, Bio-Rad) for MR. Corresponding secondary antibodies were conjugated with Alexa 488 and Cy5. DAPI was used to stain DNA. For negative controls, staining was performed without primary antibodies. Stained sections were analysed using a SP8 confocal laser scanning microscope (Leica, Mannheim, Germany).

### Statistics

Data are expressed as mean ± standard deviation. The Mann-Whitney *U* test was used to compare the variables. A *p* value ≤ 0.05 was considered significant. Statistical analysis was done using SPSS Statistics software (version 24.0.0, IBM Company, Chicago, IL, USA).

## Results

### ^68^Ga labelling of NOTA-anti-MMR Nb provided a high radiochemical purity

NOTA-anti-MMR Nb was labelled with an overall RCP of 97.2 ± 1.2% with ^68^Ga before any purification step. PD-10 purification improved the RCP up to 99.0 ± 0.6%. A specific activity of 3–4 MBq/μg was obtained.

### ^68^Ga-NOTA-anti-MMR Nb specifically binds to cultured human M2a-polarized macrophages

**Fig. 1:**
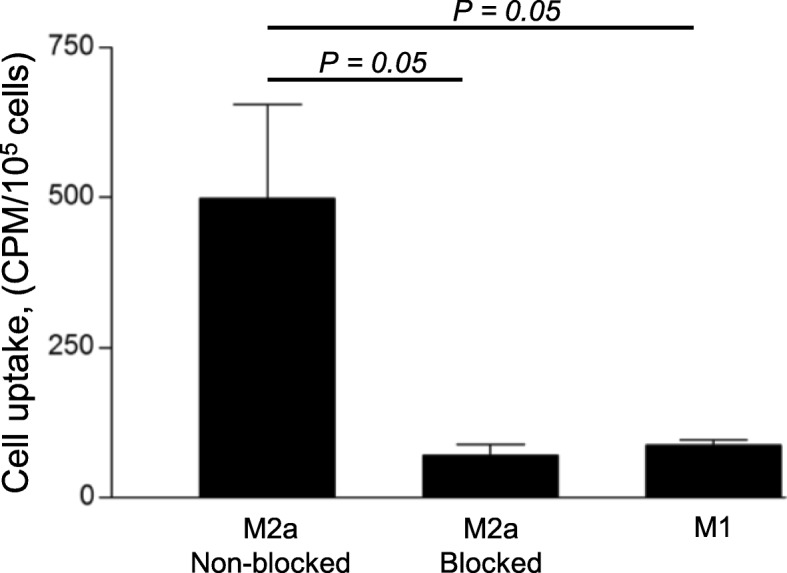
In vitro binding selectivity and specificity assay. In vitro binding selectivity and specificity of ^68^Ga-NOTA-anti-MMR Nb to MR were tested on M1- and M2a-polarized macrophages, as well as on M2a-macrophages pre-treated with a 1000-fold excess of non-labelled NOTA-anti-MMR Nb 5 min prior to the addition of 10 nM ^68^Ga-NOTA-anti-MMR Nb

Compared to M1 macrophages, IL-4-activated M2-polarized cells (M2a macrophages) displayed enhanced tracer binding. Almost 6-fold greater cell-associated radioactivity was measured for non-blocked M2a macrophages compared to the M1 subset (551 ± 170 vs 93 ± 10 CPM/10^5^ cells, respectively). Binding of ^68^Ga-NOTA-anti-MMR Nb to M2a macrophages was receptor-mediated, as demonstrated by displacement using non-radioactive compound. Blocking the receptor sites by a 1000-fold excess of non-labelled NOTA-anti-MMR Nb decreased the radiotracer uptake more than 7-fold (83 ± 20 CPM/10^5^ cells) in the M2a-blocked subset (Fig. 1).

### Rapid biodistribution and specific uptake of ^68^Ga-NOTA-anti-MMR Nb in MR^+^ organs

Data concerning the ex vivo biodistribution of ^68^Ga-NOTA-anti-MMR Nb in wildtype mice is presented in Table 1. The biodistribution data showed rapid clearance of ^68^Ga-NOTA-anti-MMR Nb from circulation via renal excretion as demonstrated by low blood values (1.4 ± 0.4% IA/g) and high kidney uptakes (144.3 ± 33.2% IA/g). Specific uptake was demonstrated in receptor-positive organs, e.g., salivary glands, liver and spleen [20, 21]. The saturation of MR by co-injection of non-labelled NOTA-anti-MMR Nb decreased the uptake of ^68^Ga-NOTA-anti-MMR Nb more than 2-fold in the above mentioned organs. The blocking effect was also observed for the heart and for fat tissue sampled from abdominal area.

**Table 1:** Biodistribution and in vivo blocking of ^68^Ga-NOTA-anti-MMR Nb in female C57BL/6 mice, 1 h p.i. The blocked group was co-injected with a 100-fold excess amount of non-labelled NOTA-anti-MMR Nb. The uptake in tissue and organs are presented as percentage of injected activity per gram of tissue (% IA/g), corrected for decay. For the gastrointestinal tract (GI) and the carcass, percentage of injected activity per gram of whole sample was calculated. Asterisks show significantly lower uptake (*p* ≤ 0.05)

Organs	Non-blocked	Blocked
Blood	1.4 ± 0.4	1.8 ± 0.5
Heart	2.3 ± 0.2	1.2 ± 0.3*
Lungs	1.4 ± 0.1	1.2 ± 0.0
Salivary glands	3.8 ± 0.8	1.8 ± 0.4*
Liver	15.8 ± 3.4	5.6 ± 1.1*
Pancreas	1.4 ± 0.1	1.3 ± 0.1
Stomach	2.0 ± 0.2	1.9 ± 0.2
Spleen	9.4 ± 1.7	4.5 ± 1.0*
Small intestine	2.1 ± 0.5	1.7 ± 0.5
Large intestine	3.8 ± 0.5	3.6 ± 0.3
Kidneys	144.3 ± 33.2*	301.6 ± 41.0
Fat	0.5 ± 0.1	0.3 ± 0.0*
Skin	1.5 ± 0.2	1.5 ± 0.0
Muscle	0.7 ± 0.1	0.6 ± 0.1
Bone	2.2 ± 0.2	2.1 ± 0.3
GI	4.1 ± 1.4	2.5 ± 0.6
Carcass	20.8 ± 1.1	19.2 ± 0.5

### Specific uptake of ^68^Ga-NOTA-anti-MMR Nb in the atherosclerotic lesions

**Fig. 2:**
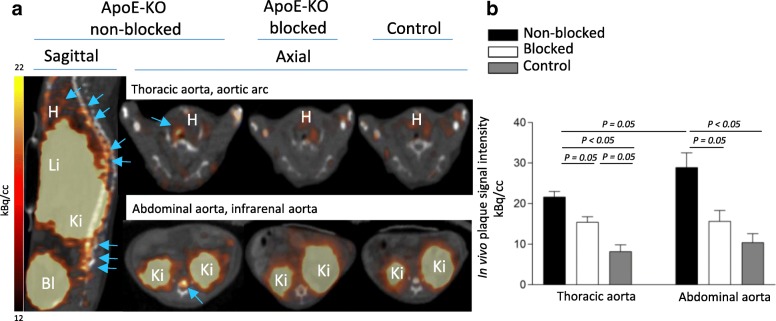
In vivo imaging and tracer uptake quantification in the atherosclerotic plaques. In vivo PET/CT images from ApoE-KO non-blocked, blocked and control mice were acquired 1 h p.i. of ^68^Ga-NOTA-anti-MMR Nb (**a**). Note the intense focal signals in the atherosclerotic plaques of ApoE-KO non-blocked mice (blue arrows). In contrast, no focal uptake was detected in the aortas of ApoE-KO mice after blocking with an excess amount of non-labelled NOTA-anti-MMR Nb or in the aortas of control mice. The upper threshold is reduced in order to reveal focal uptake, which resulted in exaggerated abdominal area signals. H heart, Li liver, Ki kidney, Bl bladder. Plaque signal intensities were quantified in thoracic and abdominal aorta using axial PET/CT images (**b**)

**Fig. 3:**
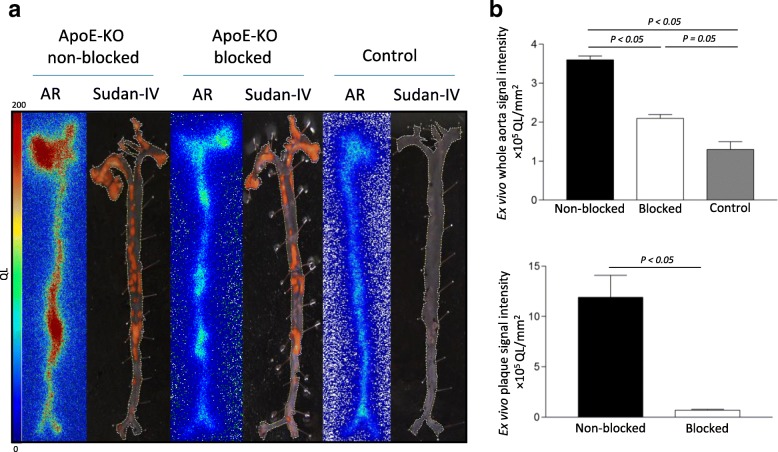
Ex vivo imaging and tracer uptake quantification in the atherosclerotic plaques. Autoradiography (AR) and corresponding Sudan-IV stained aortas excised from ApoE-KO non-blocked, blocked and control mice (**a**). Whole aorta and plaque surface areas were quantified using Image J software. Quantification of the autoradiography images expressed as intensity per unit area of whole aorta (whole aorta autoradiographic signal/whole aorta area, QL/mm^2^) (**b**). Quantification of the autoradiography images expressed as intensity per unit area of plaques ([whole aorta autoradiographic signal − normal aortic tissue autoradiographic signal]/plaque surface area, QL/mm^2^) (**c**)

The lesions in the thoracic aorta and the lower part of the abdominal aorta were clearly visualized in PET/CT images of ApoE-KO non-blocked mice. Conversely, no hotspots could be identified in the aortas of control mice or in those of ApoE-KO mice co-injected with an excess amount of non-labelled NOTA-anti-MMR Nb (Fig.2a). Morphometric data from representative images of the Sudan-IV staining revealed more lipid-laden plaques in the aortic arch and roots than in the lower abdominal aorta, which corroborates the extended autoradiographic signals, originated from the thoracic aorta, over a larger area (Fig. 3a). However, PET-derived signal intensity was slightly but significantly (*p* = 0.05) higher for the low abdominal lesions (28.9 ± 3.6 kBq/cc) compared to the plaques located in the thoracic aorta (21.6 ± 1.4 kBq/cc) (Fig. 2b). The aortas from control mice did not exhibit focal radioactivity signal and lipid staining. Mean percentage of injected dose per whole aorta dissected from ApoE-KO non-blocked mice was more than 3- and 5-fold greater than that of the ones dissected from ApoE-KO blocked and control mice, respectively. The results of quantification of the autoradiography images comparing signal intensity in whole aortas showed significantly greater uptake in aortas extracted from ApoE-KO non-blocked ((3.6 ± 0.1) × 10^5^ QL/mm^2^) compared to aortic tissue from blocked ((2.1 ± 0.1) × 10^5^ QL/mm^2^) and control mice ((1.3 ± 0.2) × 10^5^ QL/mm^2^) (Fig. 3b). Ex vivo autoradiography data showed high ^68^Ga-NOTA-anti-MMR Nb accumulation in the plaques of ApoE-KO mice ((11.9 ± 4.4) × 10^5^ QL/mm^2^). This uptake was receptor-mediated. Saturating the receptors with co-injection of non-labelled NOTA-anti-MMR Nb decreased the signal intensity in the plaques dramatically ((0.7 ± 0.1) × 10^5^ QL/mm^2^) (Fig. 3c).

### The co-expression of MR with CD68 in the lesions was confirmed by immunofluorescence staining

**Fig. 4:**
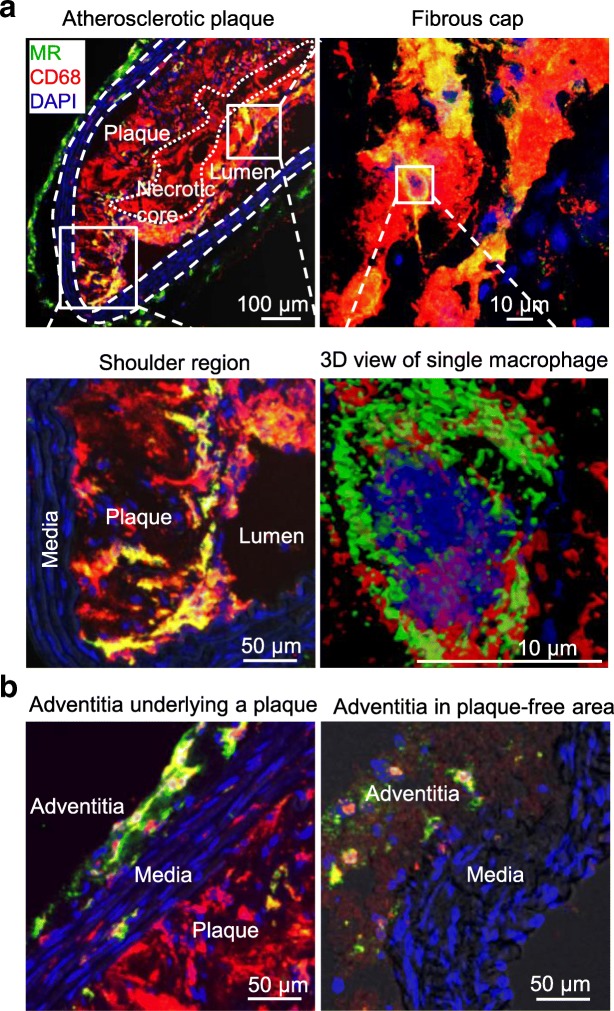
Photomicrographs of MR (green) and CD68 (red) immunofluorescence staining. Overlapping domains of expression (MR + CD68) are shown in yellow. DAPI stained nuclei are shown in blue. Some CD68^+^ macrophages mainly located in the fibrous cap and in the shoulder regions of the atherosclerotic lesions showed MR expression (**a**). The adventitia underlying atherosclerotic plaques showed more MR expression compared to the adventitia in plaque-free areas of aortas isolated from ApoE-KO and control mice (**b**)

A portion of CD68-positive (CD68^+^) macrophages located in the fibrous cap layer and shoulder region of the lesions was MR^+^ as confirmed by immunofluorescence staining (Fig. 4a). MR expression was also observed in adventitial tissue in aortas isolated from both ApoE-KO and control mice (Fig. 4b).

## Discussion

The macrophage population of atherosclerotic plaques is heterogeneous. Beside previously reported M1 and M2 macrophages, the presence of unique macrophage phenotypes has also been demonstrated in atherosclerotic lesions [6, 7]. MR^+^ macrophages were first reported by Bouhlel et al. in human carotid plaques [22]. Based on recent findings representing predominant expression of MR in fibrous cap of atherosclerotic plaques [23, 24], MR has been proposed as a potential target biomarker to identify culprit lesions. However, the exact role of alternative macrophages in atherosclerosis and their contribution to plaque vulnerability is still a matter of debate [25, 26].

Chinetti-Gbaguidi et al. have reported that IL-4-polarized CD68^+^MR^+^ macrophages express high levels of receptors involved in phagocytosis but show low capacity to ingest native and oxidized lipoproteins in vitro. The ability of these macrophage populations to clear apoptotic cells without accumulating lipids suggests that they may have beneficial roles in stabilizing atherosclerotic lesions [12]. On the other hand, the presence of M2 (CD68^+^CD163^+^) but not M1 macrophages in the fibrous cap near the rupture site of the asymptomatic thrombotic plaques in human carotid artery was reported by Mauriello et al., suggesting that alternative macrophages might also modulate the process of plaque rupture [27]. A high density of MR^+^ macrophages was also reported by Tahara et al. in high-risk plaques obtained from subjects who had experienced sudden cardiac death [28]. Furthermore, matrix metalloproteinase-9 (MMP-9, which is the most dominantly present MMP in atherosclerotic plaques) is produced by M2 rather than M1 macrophages [29, 30]. It has been reported that the rupture of carotid plaques is significantly associated with MMP-9 expression in the lesions [31]*.* As MMP-9 is capable of degrading type IV collagen [32] and triggering plaque rupture, the M2 macrophage phenotype may have a predominant role in plaque instability. In addition, the recently described MR^+^ M4 macrophages were reported to have potential pro-atherogenic roles in vulnerable plaques. They produce MMP12, an enzyme which may also be involved in the degradation of fibrous caps and hence the destabilization of atherosclerotic lesions [33–35]. Motivated by the above mentioned findings, we aimed to investigate the feasibility of imaging MR expression in atherosclerotic lesions of a murine model using radiolabelled anti-MMR Nb-based radiotracers.

In the previous study, we were not able to evaluate the relevance of the technetium-99m (^99m^Tc)-labelled anti-MMR3.49 Nb for atherosclerosis and no positive correlation was found between plaque burden and ^99m^Tc-anti-MMR3.49 Nb uptake. As confirmed by immunofluorescence staining, the absence of ^99m^Tc-anti-MMR3.49 Nb uptake in the plaques corroborated with the absence of MR expression in the lesions [36]. However, we warned for MR expression in the adventitial tissue. In the current study, however, the presence of MR^+^ macrophages, which were mainly located in the fibrous cap and in the shoulder regions of the atherosclerotic plaques, was confirmed by immunofluorescence staining. Corroborating with our previous observation, remarkable MR expression was observed in the adventitia of the aortas isolated from both ApoE-KO and control mice. The presence of macrophages in the adventitia of normal arteries has previously been reported [10, 24]. The adventitia underlying atherosclerotic plaques showed more MR expression compared to plaque-free areas of aorta segments isolated from ApoE-KO and control mice, which could be explained by the presence of adventitial cellular infiltration related to atheroma [9, 37]. Compared to ^99m^Tc, the short half-life of the ^68^Ga matches better with the fast blood clearance and target localization of the anti-MMR Nb. In addition, because of its inherently higher sensitivity and considerably better spatial resolution, PET may improve the imaging of atherosclerotic lesions compared with SPECT.

Despite the small dimension of the lesions, they were successfully visualized in the aortas of ApoE-KO mice using a small animal PET/CT scanner, 1 h p.i. of ^68^Ga-NOTA-anti-MMR Nb. In order to evaluate the specific signal from MR expression in the atherosclerotic plaques, the adventitial tissue on the aortas was removed by careful dissection for ex vivo autoradiography studies. The tracer uptake on ex vivo autoradiographic images was co-localized with Sudan-IV-positive areas corresponding to atherosclerotic plaques. Since the extent of atherosclerosis affecting the intimal surface along the aortas was not the same in all animals, the autoradiographic signals were normalized to the area of plaques, measured after Sudan-IV staining.

MR has been adopted as a biomarker to identify rupture-prone atherosclerotic plaques, and different targeted nuclear imaging probes have been developed with the aim of visualizing MR expression in the lesions. The feasibility of ^18^F-labelled D-mannose (2-deoxy-2-[^18^F]fluoro-d-mannose, ^18^F-FDM) for imaging of atherosclerotic lesions was reported by Tahara et al. [28]. They demonstrated that ^18^F-FDM uptake is not inferior to that of ^18^F-FDG for imaging of plaque inflammation [28]. The ^68^Ga-labelled NOTA-coupled mannosylated human serum albumin was reported by Kim et al. as a radiotracer for non-invasive detection of M2 macrophages in vulnerable atherosclerotic plaques [38]. Recently, we demonstrated the feasibility of ^111^In-tilmanocept for non-invasive in vivo targeting of plaque inflammation in ApoE-KO mouse model [39]. Although the success of these radiotracers for in vivo visualization of atherosclerotic plaques has been well documented—due to the fact that mannose is an isomer of glucose, ^18^F-FDM as well as other above mentioned mannosylated radiotracers may be taken up by all macrophages (similarly to ^18^F-FDG) and thus may be unsuitable for discrimination between different phenotypes. In the present study, however, our objective was to assess the potential of an anti-MMR Nb for specific targeting of MR^+^ macrophages and in vivo imaging of atherosclerotic plaques in ApoE-KO mice.

Radiotracers based on anti-MMR Nbs have been well evaluated for imaging tumour-infiltrating macrophages [13, 14] and joint inflammation in rheumatoid arthritis animal models [15]. The specificity of the tracers to MR has also been confirmed in MR-KO mice [13, 14]. ^68^Ga-NOTA-anti-MMR Nb has also been thoroughly investigated in a rabbit model of atherosclerosis [40]. Using a clinical PET/MR scanner, a gradual increase in signal intensity was observed in the aortas of atherosclerotic rabbits as disease progressed, confirming translatability to other animal species.

This study has some limitations. Regardless of its sufficient expression by macrophages located in atherosclerotic plaques for in vivo imaging, MR is also expressed by some cells in adventitia which causes a non-negligible background signal when using ^68^Ga-NOTA-anti-MMR Nb. The presence of MR^+^ cells in several abdominal area organs might also have hampered the in vivo detection of radiotracer uptake in the abdominal aorta, due to the small size of the mouse body. Although we were able to evaluate the relevance of MR targeting with ^68^Ga-NOTA-anti-MMR Nb in ApoE-KO mouse model of atherosclerosis, whether ^68^Ga-NOTA-anti-MMR Nb also accumulates in complex human atherosclerotic plaques needs to be validated in future studies.

## Conclusion

More clinically relevant animal models are necessary to study the exact role of distinct macrophage phenotypes and their impact on plaque (in)stability. Accordingly, the contribution of MR^+^ macrophages in the evolution and vulnerability of atherosclerotic lesions and the predictive value of MR imaging for vulnerable patients should be further investigated. Nevertheless, as inflammation is a major event in atherosclerosis, imaging of macrophages, and in particular their phenotypes, is an attractive approach for plaque imaging and therapy.

Our study indicates that ^68^Ga-NOTA-anti-MMR Nb is a promising candidate as a probe for imaging MR expression in vivo. The presented data confirm that small dimension atherosclerotic plaques can be efficiently targeted and visualized using ^68^Ga-NOTA-anti-MMR Nb in ApoE-KO mouse model.

## Abbreviations

% IA/g: Percentage of injected activity per gram of tissue
ApoE-KO: Apolipoprotein E-knockout
i.v.: Intravenous
MMR: Macrophage mannose receptor
MR: Mannose receptor
Nb: Nanobody
PBMC: Peripheral blood mononuclear cell
PET/CT: Positron emission tomography/computed tomography
p-SCN-Bn-NOTA: 2-S-(4-Isothiocyanatobenzyl)-1,4,7-triazacyclononane-N,N′,N″-triacetic acid
QL: Quantum level
ROI: Region of interest
